# 163. Can Machine Learning Guide Antibiotic Initiation for Lower Respiratory Tract Infections?

**DOI:** 10.1093/ofid/ofad500.236

**Published:** 2023-11-27

**Authors:** Sarah Tang, Daniel Chang, De Zhi Chin, Maciej Piotr Chlebicki, Shimin Jasmine Chung, Lai Wei Lee, Winnie Lee, Peijun Yvonne Zhou, Andrea Kwa

**Affiliations:** Singapore General Hospital, Singapore, Not Applicable, Singapore; SingHealth, Singapore, Not Applicable, Singapore; Singapore General Hospital, Singapore, Not Applicable, Singapore; Singapore General Hospital, Singapore, Not Applicable, Singapore; Singapore General Hospital, Singapore, Not Applicable, Singapore; Singapore General Hospital, Singapore, Not Applicable, Singapore; Singapore General Hospital, Singapore, Not Applicable, Singapore; Singapore General Hospital, Singapore, Not Applicable, Singapore; Singapore General Hospital, Singapore, Not Applicable, Singapore

## Abstract

**Background:**

Accurate diagnosis of acute bacterial lower respiratory tract infections (LRTI) is often elusive. The lack of diagnostic gold standard, exacerbated by overlapping syndrome with non-infective/non-bacterial respiratory conditions, have contributed to antibiotic over-prescribing. This in turn drives drug resistance, healthcare costs and patient morbidity. We explored feasibility of developing a

classification model to identify instances where antibiotics should be initiated or withheld when bacterial LRTI is suspected.

**Methods:**

All inpatients in Singapore General Hospital during Y2019-2020 and had ICD-10 codes for LRTI or mimicking conditions were included. Only cases with ICD-10 codes for LRTI and manually assessed to be true by the hospital antimicrobial stewardship unit (ASU) were labelled as ground truth “Yes” (i.e., antibiotics should be initiated). Cases with ICD-10 codes for other conditions were labelled as ground truth “No” (i.e., antibiotics could be withheld). Patient information (e.g., clinical notes, radiology reports, vital signs) were extracted from our hospital’s electronic data repository. Gradient boosting algorithm was applied for model building and evaluated on a 20% held-out test set. A sub-analysis on incorrect classification was also performed.

**Results:**

There were a total of 24,399 episodes. After exclusion by ASU review and under-sampling, the ground truth “yes” and “no” groups had 2,887 and 4,888 cases respectively. The model achieved a good performance of 80% accuracy, 83% sensitivity and 79% specificity, corresponding to an AUC of 0.89. Chest imaging findings, procalcitonin, c-reactive protein, cough, neutrophil count and oxygen saturation at start of episode were the 5 most important features. Incorrect classifications had lower confidence scores, and were more likely to occur among those with severe illness or radiological reports that were not suggestive of LRTI.

**Conclusion:**

We developed a machine learning model to support decision-making on antibiotic initiation for suspected bacterial LRTI. Further work is needed to optimise this model and evaluate its impact in clinical practice.

**Disclosures:**

**All Authors**: No reported disclosures

